# Supplementary material for: Usp16 modulates Wnt signaling in primary tissues through Cdkn2a regulation
Source: Sci Rep. 2018 Nov 30;8:17506. doi: 10.1038/s41598-018-34562-w (PMC6269430; doi:10.1038/s41598-018-34562-w)
Supplement: Supplementary file 1 — Supplementary figures [file 41598_2018_34562_MOESM1_ESM.pdf]

SUPPLEMENTARY INFORMATIONS

Title: **Usp16 modulates Wnt signaling in primary tissues through Cdkn2a regulation**

Authors: Maddalena Adorno, Benedetta Nicolis di Robilant, Shaheen Sikandar, Veronica Haro Acosta, Jane Antony, Craig Heller, Michael F. Clarke

Supplemental Figure S1

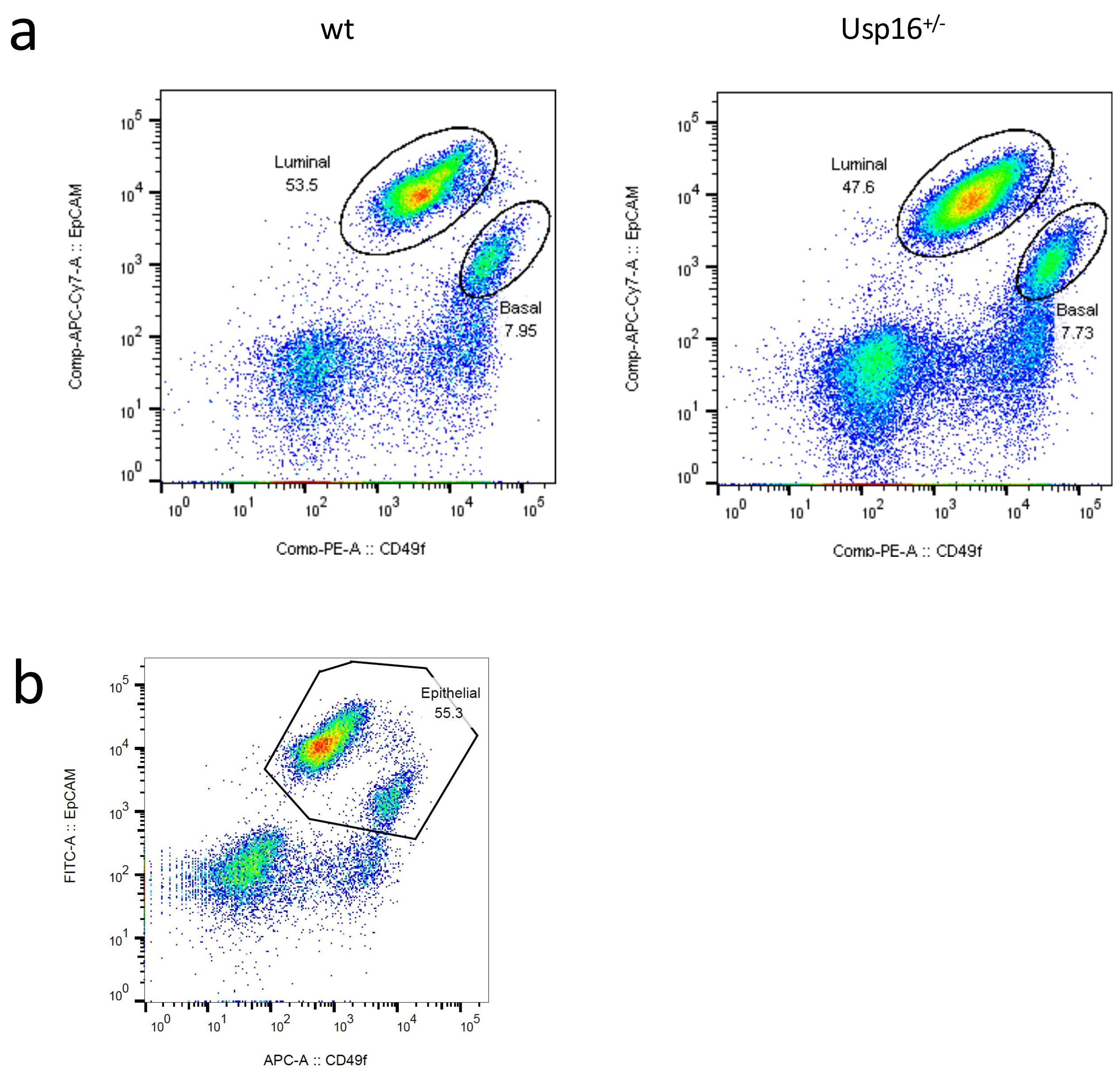

(A) Representative FACS plots of Lin$^-$ (Ter119$^-$ CD45$^-$CD31$^-$) mammary glands for the indicated genotypes. There is no difference in the basal/luminal ratio between wt and Usp16$^{+/-}$ mice. **(B)** Representative FACS plots of Lin$^-$ mammary epithelial cells showing the gating strategy used to isolate mammary epithelial cells based on the expression of CD49f and EpCAM.

Supplemental Figure S2

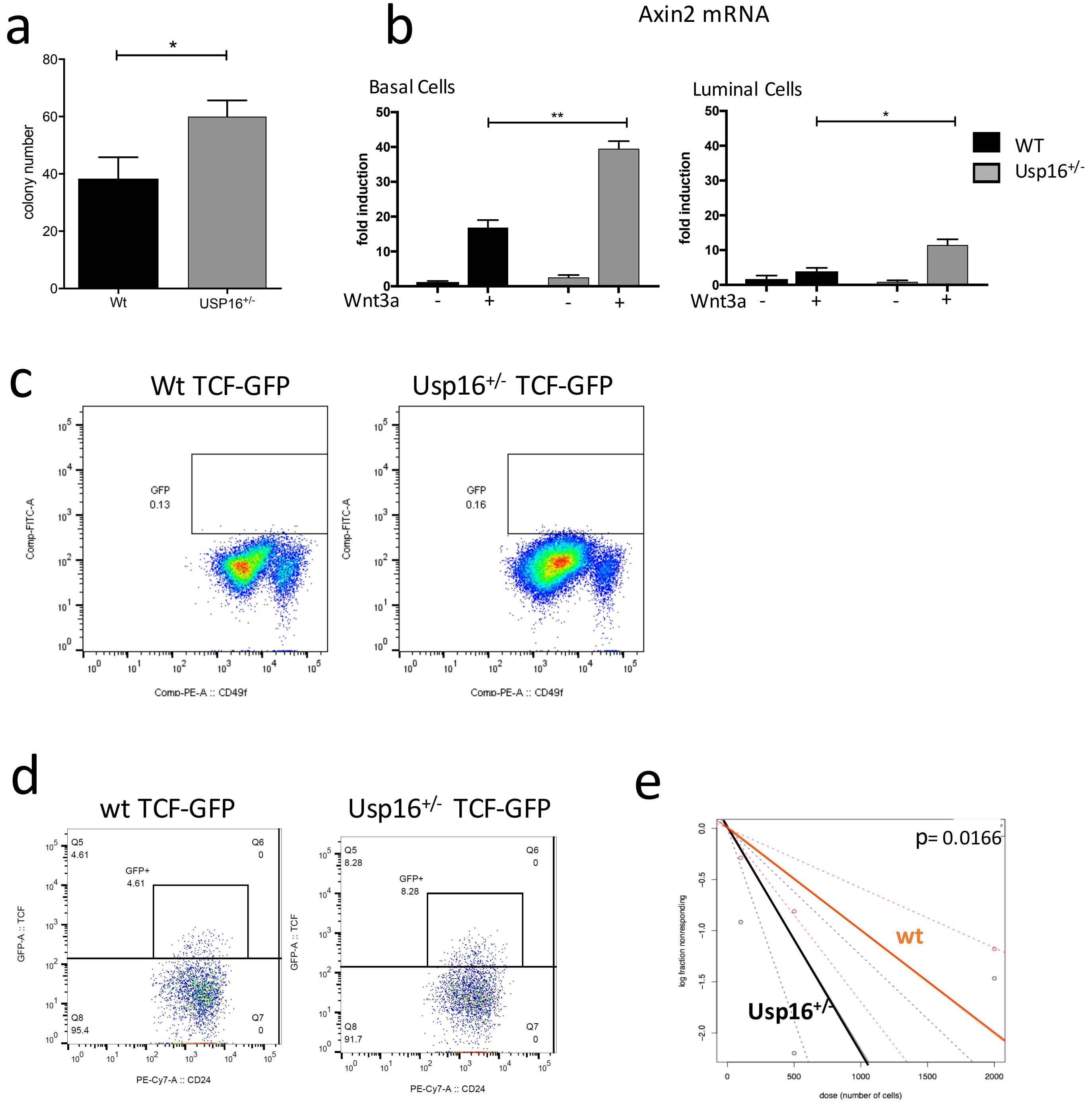

(A) Usp16$^{+/-}$ sorted epithelial cells form more colonies *in vitro* then wt cells when cultured with recombinant Wnt3a (100ng/ml) in the absence of a feeder layer (n=3). **(B)** Usp16$^{+/-}$ sorted basal and luminal cells show an increased induction of Axin-2 mRNA levels 16 hours after Wnt3A stimulation (50ng/ml). Three independent experiments were performed. **(C)** Representative FACS plots of mammary epithelial cells derived from TCF/LEF-H2B/GFP mice. GFP^{+} cells were not detectable. **(D)** After one in vitro passage, the percentage of GFP^{+} cells was increased in Usp16$^{+/-}$ TCF-GFP mice compared to wt. **(E)** Limiting dilution analysis (ELDA) shows an increased frequency of mammary repopulating cells in Usp16$^{+/-}$ compared to wt breast tissue. Four rounds of transplants were performed. ELDA graph was generated through the software available at www.bioinf.wehi.edu.au/software/elda.

Supplemental Figure S3

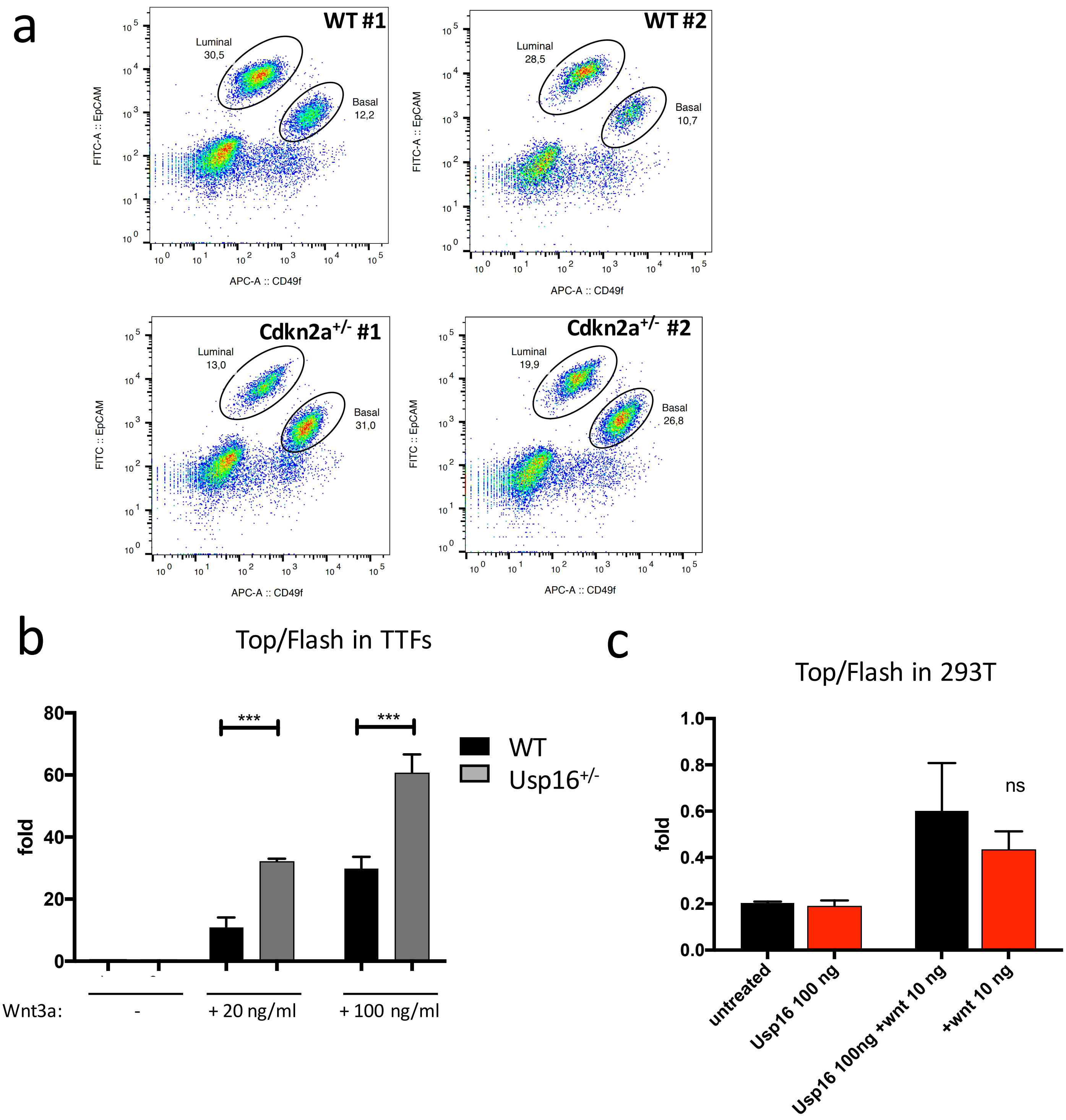

(A) Representative FACS plots of Lin^- mammary epithelial cells for the indicated genotypes. CD49f and EpCAM allow the identification of the basal and luminal populations. **(B)** TTFs derived from animals with the indicated genotypes were analyzed for their ability to activate the Top-flash reporter at two different doses of Wnt3a (18 hours of treatments). Renilla was used to normalize luciferase data. $Usp16^{+/-}$ cells activate Top Flash more efficiently then wt cells. Experiments were repeated three times. **(C)** HEK293T cells were transfected with a plasmid overexpressing Usp16 or a control plasmid. Cells were then tested for the activation of the Top-flash reporter in the absence or presence of 10 ng/ml of Wnt3a. No significant difference was observed after overexpression of Usp16. HEK293T cells do not have an intact Cdkn2a signaling.

Supplemental Figure S4

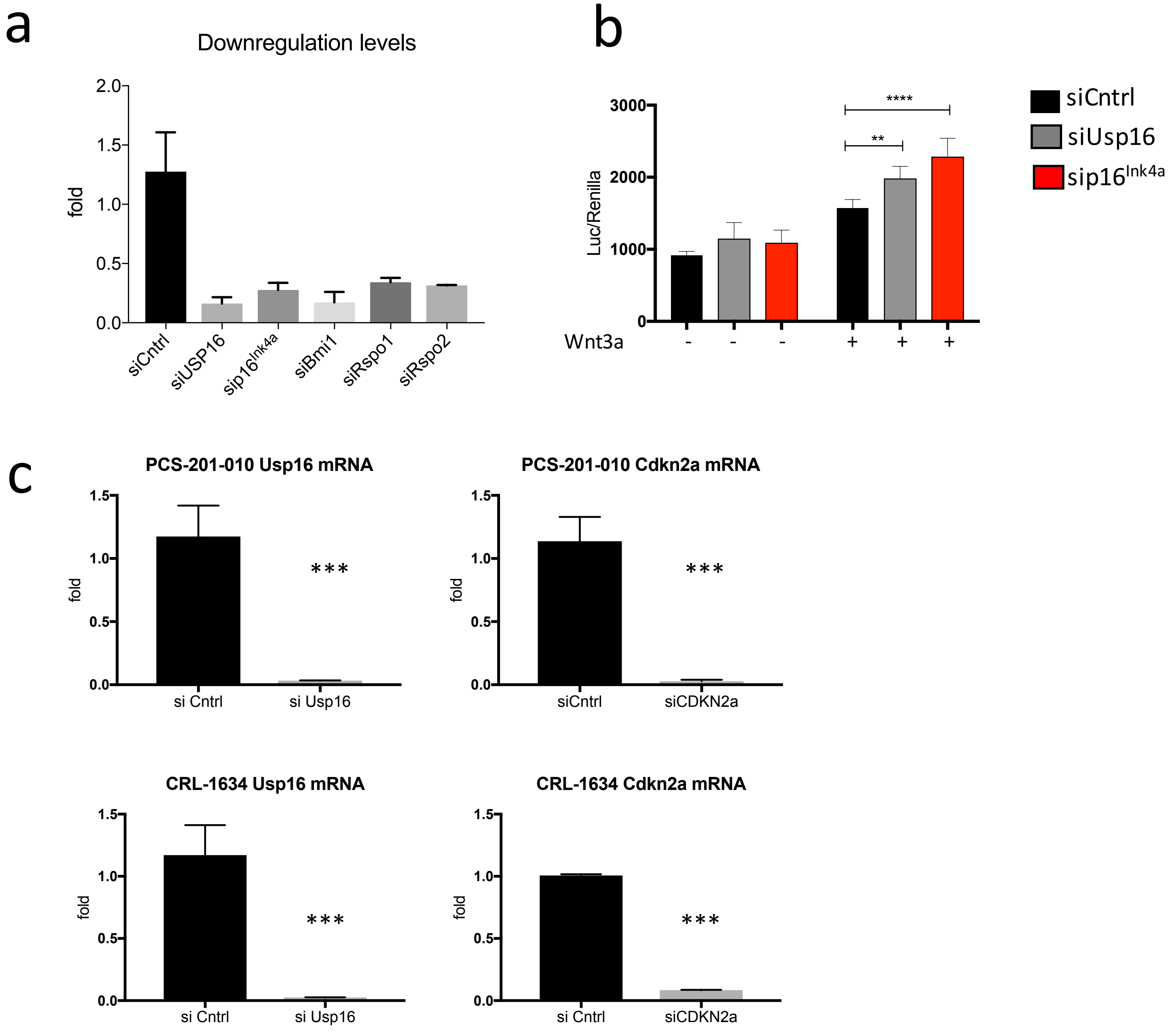

(A) MEF cells were tested for levels of RNA downregulation after transient transfection of siRNA. All siRNAs efficiently abrogate target's gene expression ($p<0.001$) **(B)** MEFs were transiently transfected with two individual siRNAs targeting Usp16 or $p16^{Ink4a}$. Activation of 6KD Wnt reporter is shown, normalized by Renilla expression. Cells were treated with 20ng.Two different human primary cultures were used (CRL-1634 and PCS-201-010) to test AXIN2 mRNA regulation in response to Wnt3a treatment ((Figure 4C). **(B)** Downregulation of USP16 and CDKN2a was tested by real time PCR in untreated cells. siRNAs efficiently abrogate target gene expression in both cell lines ($p<0.001$).

Supplemental Figure S5

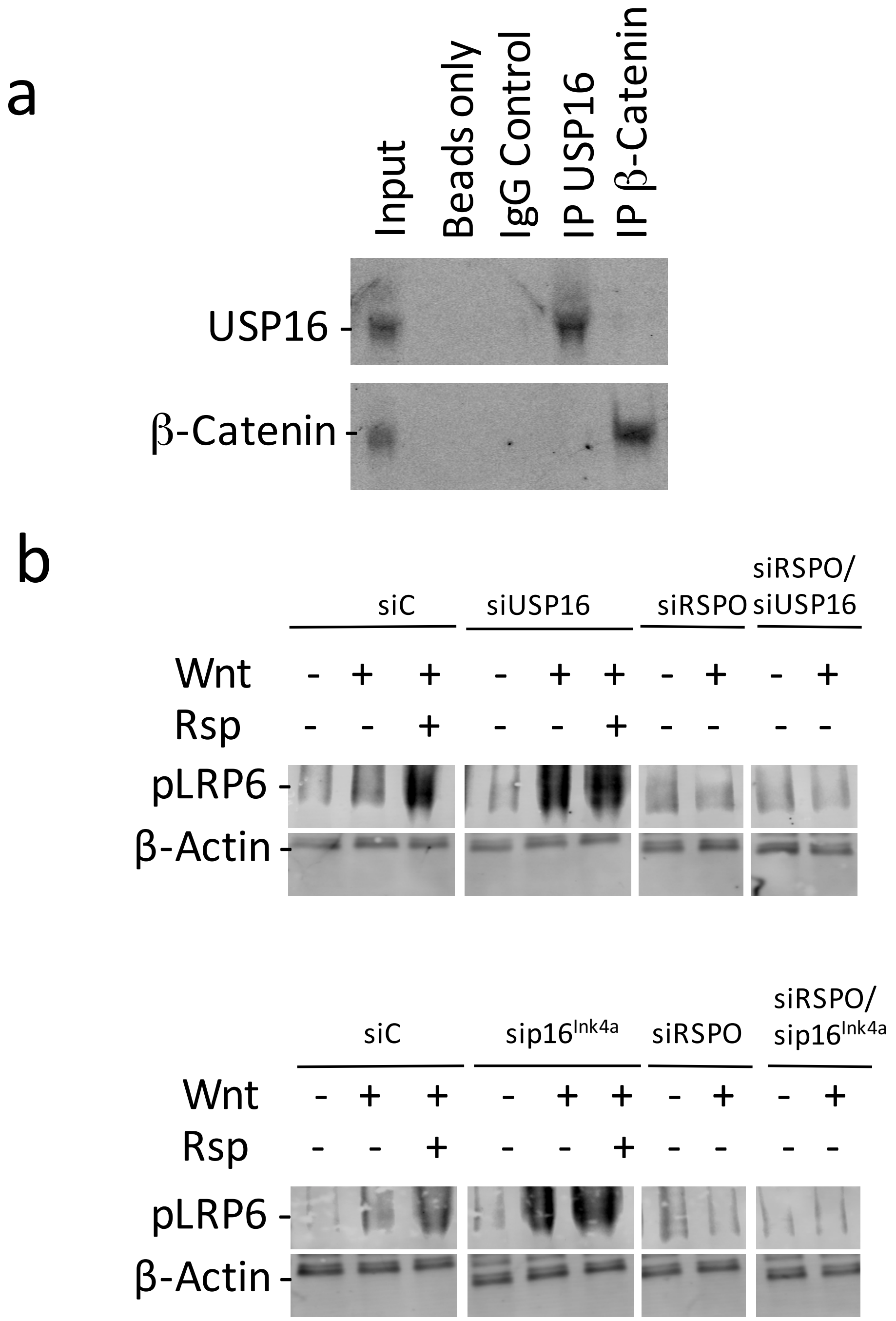

(A) Immunoprecipitation for Usp16 in MEF cells at passage p2 does not show any binding for βcatenin. Immunoprecipitation for βcatenin doesn't show any binding for Usp16. **(B)** Western blot of MEF cells after transient knockdown of Usp16, $p16^{Ink4a}$ and/or Rspo1/Rspo2. Usp16 or $p16^{ink4}$ knockdown potentiates pLRP6 phosphorylation even without Rspo treatment. Knockdown of Rspo1 and Rspo2 abrogates this effect and it is not rescued by Usp16 or $p16^{Ink4a}$ knockdown.

Supplemental Figure S6

a

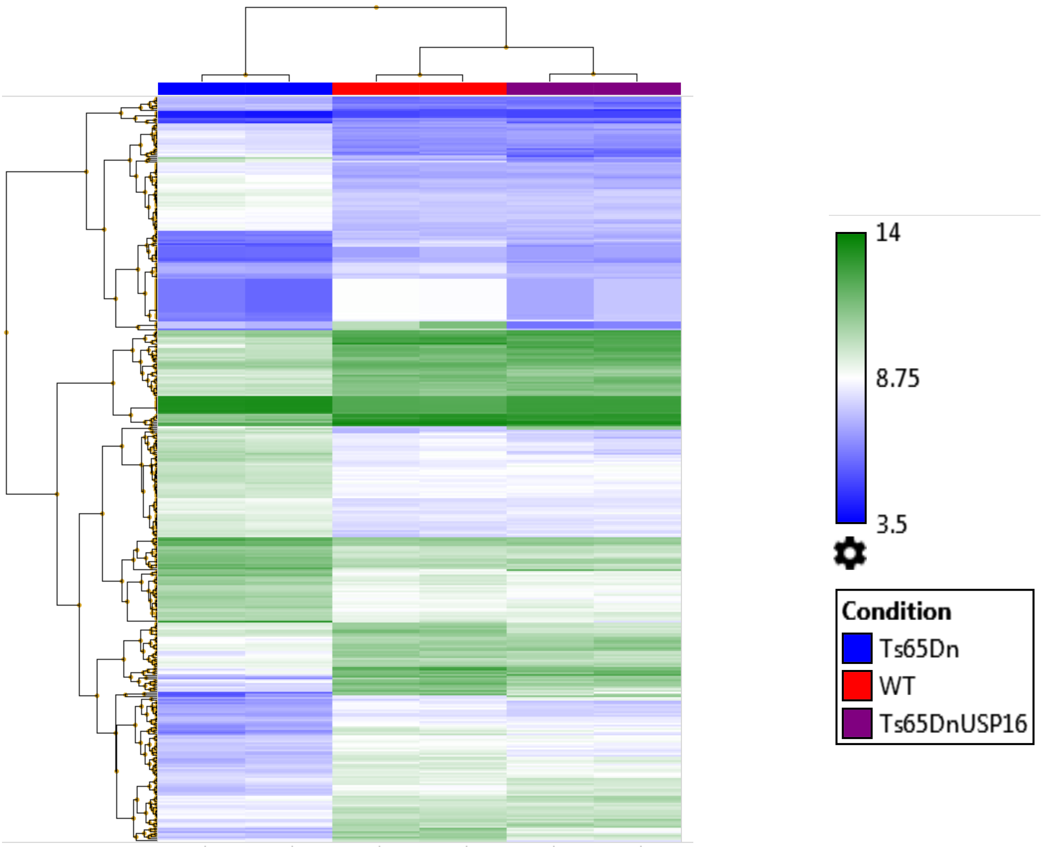

(A) Unsupervised hierarchical clustering of RNA expression profiles derived from TTFs with the indicated genotypes. Condition F-test <0.001 for wt vs Ts65Dn samples. Ts65Dn/Usp16$^{+/-}$ samples cluster with wt, showing how the expression profile of Ts65Dn cells is strongly dependent on Usp16 trisomy.
