# Supplementary material for: Usp16 modulates Wnt signaling in primary tissues through Cdkn2a regulation
Source: Sci Rep. 2018 Nov 30;8:17506. doi: 10.1038/s41598-018-34562-w (PMC6269430; doi:10.1038/s41598-018-34562-w)
Supplement: Supplementary file 2 — Legends for Supplementary Tables 1 and 2 [file 41598_2018_34562_MOESM2_ESM.docx]

Legends for Supplementary dataset 1-2

**Title**: **Usp16 modulates Wnt signaling in primary tissues through Cdkn2a regulation**

**Authors:** Maddalena Adorno^1^, Benedetta Nicolis di Robilant^1^, Shaheen Sikandar^1^, Veronica Haro Acosta^1,2^, Jane Antony^1^, Craig Heller^3^, Michael F. Clarke^1*^

**Table T1**

Table of 413 genes used to define the “Wnt signature”. The list was derived from the MGI gene ontology annotation (<http://www.informatics.jax.org)>.

**Table T2**

List of genes derived from the “Wnt signature” and differentially expressed between wt and Ts65Dn. This list was used for the Wnt-specific clustering (fold change <-1.5 or >1.5 and a conditional F-test <0.05).
